# DeepPulmoTB: A benchmark dataset for multi-task learning of tuberculosis lesions in lung computerized tomography (CT)

**DOI:** 10.1016/j.heliyon.2024.e25490

**Published:** 2024-02-07

**Authors:** Zhuoyi Tan, Hizmawati Madzin, Bahari Norafida, Yang ChongShuang, Wei Sun, Tianyu Nie, Fengzhou Cai

**Affiliations:** a Faculty of Computer Science and Information Technology, Universiti Putra Malaysia, Serdang, 43400, Malaysia; b Department of Radiology, Universit Putra Malaysia, 43400 Serdang, Selangor, Malaysia; c College of Computer Science, Chongqing University, Chongqing 400030, China; d Electronics and Computer Science, University of Southampton, Southampton, SO17 1BJ, UK

**Keywords:** Multi-task learning, Segmentation, Classification, CT tuberculosis imaging

## Abstract

Tuberculosis (TB) remains a significant global health challenge, characterized by high incidence and mortality rates on a global scale. With the rapid advancement of computer-aided diagnosis (CAD) tools in recent years, CAD has assumed an increasingly crucial role in supporting TB diagnosis. Nonetheless, the development of CAD for TB diagnosis heavily relies on well-annotated computerized tomography (CT) datasets. Currently, the available annotations in TB CT datasets are still limited, which in turn restricts the development of CAD tools for TB diagnosis to some extent. To address this limitation, we introduce DeepPulmoTB, a CT multi-task learning dataset explicitly designed for TB diagnosis. To demonstrate the advantages of DeepPulmoTB, we propose a novel multi-task learning model, DeepPulmoTBNet (DPTBNet), for the joint segmentation and classification of lesion tissues in CT images. The architecture of DPTBNet comprises two subnets: SwinUnetR for the segmentation task, and a lightweight multi-scale network for the classification task. Furthermore, to enhance the model's capacity to capture TB lesion features, we introduce an improved iterative optimization algorithm that refines feature maps by integrating probability maps obtained in previous iterations. Extensive experiments validate the effectiveness of DPTBNet and the practicality of the DeepPulmoTB dataset.

## 1 Introduction

Over the past 130 years, tuberculosis (TB) has persisted as a formidable global health challenge, capturing extensive attention in the field of public health due to its high incidence and mortality rates. Currently, early disease diagnosis plays a crucial role in addressing this challenge. By enabling timely and effective treatment interventions, early diagnosis improves treatment outcomes and patient survival rates [6].

In recent years, computer-aided diagnosis (CAD) tools have played an increasingly important role in enhancing the efficiency of disease diagnosis [40], [36], [12]. Deep-learning models, a key component of CAD tools, have gained significant popularity because of their remarkable ability to recognize complex lesion tissues. However, a notable drawback of deep-learning models is that they rely on extensive training data to achieve optimal performance. This limitation hinders their application in domains characterized by limited training data availability, particularly in the realm of automatically and accurately identifying TB lesion tissue in lung computerized tomography (CT). In this field, due to the complex shape and outline of tuberculosis lesions, deep-learning models often require a large amount of fine-grained annotation information to achieve better performance.

**Figure 1:**
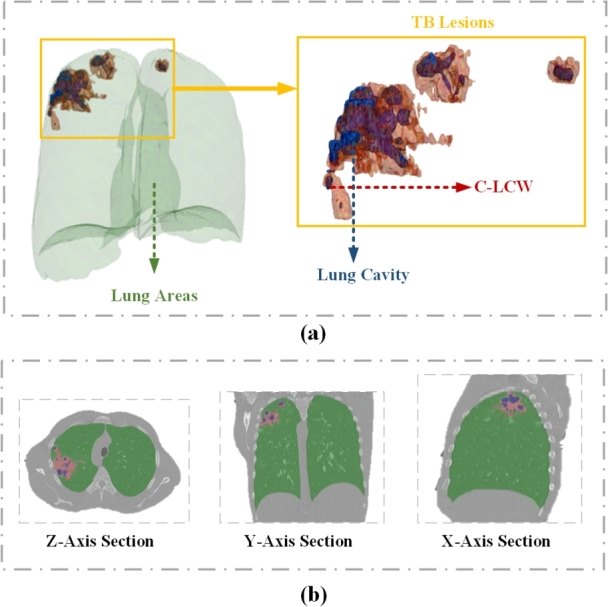
Visualization of mask data for each category in the DeepPulmoTB dataset: (a) Spatial 3D representation of the mask data structure. (b) Cross-sectional results (X, Y, Z planes) of the sample depicted in (a). C-LCW represents the unified category of Consolidation and Lung Cavity Wall.

In this paper, we construct a CT multi-task learning dataset specifically designed for TB diagnosis, DeepPulmoTB. It is a comprehensively annotated multi-task learning dataset that encompasses both segmentation and classification tasks. Firstly, the segmentation task encompasses three essential TB diagnostic categories (as shown in Fig. 1): lung cavity, C-LCW, and lung areas. Secondly, the classification task includes descriptive information about the lung cavity size and quantity.

To demonstrate the practical utility of DeepPulmoTB, we introduce an innovative deep-learning framework, termed DeepPulmoTBNet (DPTBNet). This architecture can accurately perform the segmentation of TB lesion tissues in CT images and conduct attribute classification of lung cavity tissue. Through an exhaustive array of experiments, we have empirically validated the efficacy and robustness of the proposed DPTBNet. The experimental results show its remarkable aptitude in accurately delineating pivotal TB lesion components, including lung cavities and C-LCW, while simultaneously providing precise characterizations of lung cavity attributes. Our main contributions in this work are summarized as follows:

1. We have created a dataset called DeepPulmoTB, specifically designed for CT TB multi-task learning. To our knowledge, DeepPulmoTB is the first multi-task dataset available for joint training of TB lesion tissue classification and segmentation tasks. In addition, DeepPulmoTB also provides the first dataset available for multi-category tuberculosis lesion segmentation.
2. We propose a novel multi-task learning model called DeepPulmoTBNet (DPTBNet), aimed at performing joint segmentation and classification of lesion tissues in CT images.
3. We introduce an improved iterative optimization algorithm that optimizes feature maps by fusing probability maps from previous iterations to improve the ability to detect TB lesion features.
4. Extensive experiments confirm DPTBNet's effectiveness and DeepPulmoTB's practicality.

## 2 Related work

### 2.1 Tuberculosis CT lesions recognition dataset

Table 1 summarizes the annotation categories of existing CT tuberculosis lesions recognition datasets. From the table, we observed that previous datasets have relatively limited segmentation categories, for example, including just the lung cavities class. Additionally, some datasets primarily use object detection bounding boxes to annotate TB lesions [20], [22]. However, this form of annotation only provides a rough estimate of the approximate location of the lesion tissue, without capturing precise details regarding the shape of specific lesions, thereby limiting the accurate identification of lesion tissues to some extent. In contrast, the DeepPulmoTB dataset annotates key identification information for multiple TB diagnoses (lung cavities, C-LCW, and lung area) and integrates them into a single semantic segmentation dataset, which helps fill the gap of the lack of diverse and comprehensive datasets in the field of multi-category TB semantic segmentation research.

**Table 1:** Comparison of DeepPulmoTB with other existing TB lesions recognition datasets. CLEF22TB represents imageCLEF2022 TB dataset [16]. CLEF20-21TB represents imageCLEF2019 - 2021 TB series dataset [15], [21], [14]. The terms (Seg) and (Det) refer to segmentation and detection tasks, respectively.

	CLEF19-21TB	[39]	[22]	CLEF22TB	[41]	DeepPulmoTB
C-LCW	N/A	✓(Seg)	✓(Det)	N/A	N/A	✓(Seg)
Lung Cavity	N/A	N/A	✓(Det)	✓(Det)	✓(Seg)	✓(Seg)
Lung Areas	✓(Seg)	N/A	N/A	✓(Seg)	N/A	✓(Seg)
Multi-Category Segmentation	N/A	N/A	N/A	N/A	N/A	✓

### 2.2 Tuberculosis lesions descriptive analysis dataset

Tuberculosis lesion descriptive analysis datasets typically encompass descriptive information about attributes associated with tuberculosis lesions. One of the most commonly used datasets for the analysis of CT TB lesions is the “TB Portals” provided by the National Institutes of Health (NIH). This dataset is intended exclusively for the study of drug-resistant tuberculosis. The “TB Portals” dataset [31] contains descriptive analysis information on the lesion tissues of tuberculosis patients that includes factors such as lung cavity size, total number of cavities, transmission status, and more. In addition, there is the ImageCLEF TB series dataset [15], [16], [21], [14]. This series of datasets provides image analysis and diagnostic information about tuberculosis and its lesions. This information includes an assessment of the severity of pulmonary tuberculosis, classification of tuberculosis lesion types, etc.

### 2.3 Tuberculosis image segmentation

In previous studies, many segmentation methods for CT tuberculosis images were based on traditional image processing techniques. Some commonly traditional used methods for TB lesion segmentation include thresholding [10], [26], region growing [3], and edge detection [29], [42]. While these methods can achieve satisfactory results to some extent, the robustness and accuracy of traditional image processing techniques may be limited due to the diverse shapes and sizes of TB lesions, as well as the presence of noise and artifacts in the images. Additionally, for the segmentation of TB lung regions, some commonly traditionally used methods include clustering [8], non-rigid image registration [34] and watershed transform [32]. However, these methods may struggle to achieve precise segmentation when dealing with fuzzy boundaries of lung airways and lesions. Moreover, these traditional lung region segmentation methods may be more sensitive to CT images with poor quality, necessitating consideration of how to handle low-quality images.

In recent years, convolutional neural network (CNN)-based methods have been applied to the segmentation of TB lesions and lung regions in CT tuberculosis images [39], [41], [28]. For TB lesion segmentation, [39] proposed an automatic tuberculosis lesion segmentation method based on the U-Net neural network. Additionally, they combined the Canny edge detection algorithm with the network to achieve more accurate TB lesion boundaries. [41] proposed a novel RNN and graph convolutional network (R2GCN) method that integrates bidirectional recurrent networks (BRN) and graph convolutional networks (GCN) modules. [28] proposed a Multi Res U-Net segmentation model for segmenting the pulmonary tuberculosis-affected region. [38] proposed to use the U-Net and attention mechanism to build an Attention U-Net network model for feature extraction and segmentation of labeled tuberculosis CT images to achieve lesion segmentation and lesion labeling of unlabeled tuberculosis CT image data.

Furthermore, for the segmentation of lung regions in CT images, [44] proposed an Attention Multi-scale Fusion UNet (AMF-UNet) method based on convolutional neural networks. In this work, the authors designed a dual attention mechanism in the model to preserve crucial spatial information of the damaged lung regions and improve segmentation accuracy. Moreover, for non-tuberculosis lung region segmentation, [19] proposed a deep learning architecture named Residual U-Net, and they incorporated a false-positive removal algorithm for lung CT segmentation. They believed that learning from deeper layers of the network could extract more discriminative feature representations compared to shallow networks. [13] proposed an automatic lung segmentation method in CT images using a convolutional neural network (CNN) Mask R-CNN specifically designed for lung region mapping, combined with supervised and unsupervised machine learning methods.

However, the limitations of these methods lie in their yet to directly address challenges posed by the diverse shapes and sizes of TB lesions, as well as the presence of fuzzy image boundaries in tuberculosis CT images. Addressing these challenges through further research and improvement of multi-task learning methods will contribute to enhancing the accuracy of the segmentation and classification of tuberculosis CT images.

### 2.4 Tuberculosis image classification

Traditional methods for classifying pulmonary tuberculosis in CT images typically rely on manual feature extraction and handcrafted rules, including morphological operations [9], texture feature extraction [7], threshold segmentation methods [30], and machine learning methods [4], [27]. [9] proposed a morphology-based lung graph model that partitions the lung into regions represented as nodes in the graph and defines weighted edges between adjacent regions based on the distance between their 3D texture descriptors. [7] presented a texture-based lung graph model to describe features of different types of TB patients, constructing a lung graph where nodes represent lung regions and edges encode relationships between internal textures of the regions. [1] introduced two texture-based analysis methods for TB lesion classification: a 3D modeling approach and a slice-based extraction method. These methods were used to generate feature values from CT scans and tested with different classifiers. [4] used various machine learning classification methods, including multi-class support vector machine (SVM) with radial basis function (RBF) kernel, k-nearest neighbor (kNN) algorithm, and multi-layer perceptron classifier with different parameter settings, to analyze image features extracted by binarization methods in order to predict TB severity scores and levels.

The above classification methods heavily depend on the accuracy of manually extracted features, making them less applicable to other classification tasks. In contrast, deep learning techniques have powerful feature extraction and processing capabilities, allowing them to overcome the limitations of traditional classification methods. [2] proposed a model called DAvoU-Net, which combines CNN and RNN deep learning architectures for lung CT image TB segmentation and severity evaluation. This model incorporates dense connections and multi-scale context to enhance output mappings and improve feature sizes. [25] introduced the use of feature maps to generate context-aware features and developed a novel context-aware graph neural network called TBNet for detecting TB in chest CT images.

Furthermore, [33] proposed a deep learning-based TB-type classification method. Firstly, they pre-trained a generative adversarial network (GAN) on different CT scan datasets, with the discriminator serving as the pre-trained model to gradually learn how to distinguish real from fake samples. Then, they fine-tuned the discriminator on the primary dataset to more accurately predict the TB type for each infected chest CT scan.

### 2.5 Joint classification and segmentation

In the field of multi-task learning for the classification and segmentation of pulmonary tuberculosis (TB) CT images, there are currently limited methods available. However, the concept of multi-task learning has been applied to TB X-rays and other medical applications. For TB X-ray images, [17] proposed a novel TB-UNet model that utilizes dilated fusion blocks (DF) and attention blocks (AB) for accurate segmentation of lung regions. Additionally, they introduced TB-DenseNet, a model based on five dual convolution blocks, DenseNet-169 layer, and a feature fusion block, for the precise classification of TB images. [2] also presented a novel image segmentation method called DAvoU-Net, which combines multi-scale residual blocks (MRB) and receptive dense connections (RDC). The feature learning approach of this model is unique, as it transforms the two-dimensional weight parameters of pre-trained neural networks into three-dimensional weights through rotation operations, used for initializing 3D convolutional neural networks (CNNs) for deep feature extraction. This technique overcomes the limitations of using 2D weighted pre-trained networks as transfer learning techniques on 3D-CNNs, generating deep image feature vectors with sufficient discriminative ability for representing TB severity assessment. [11] proposed a new deep learning model that combines 3D CNNs with a novel loss function introduced by [18], which plays a crucial role in multi-task learning, contributing to model optimization and achieving better performance.

Multi-task learning is also applicable to other medical images. [45] proposed a novel multi-task learning framework for joint segmentation and classification of tumors in Automated Breast Ultrasound (ABUS) images. The proposed framework consists of two sub-networks: an encoder-decoder network for segmentation and a lightweight multi-scale network for classification. [35] proposed an end-to-end multi-task deep learning framework for automatic skin lesion analysis. This framework can simultaneously perform skin lesion detection, classification, and segmentation tasks.

However, the aforementioned multi-task learning methods cannot be directly applied to the joint segmentation and classification of TB CT data. Firstly, in TB CT images, the complex shape of lesion tissues and the unclear boundaries make the segmentation of TB lesions more challenging compared to other application domains. Secondly, in the classification task of TB, different types of lesions may exhibit similar CT appearances, leading to difficulties in classification. Therefore, for the joint segmentation and classification of TB CT data, a specific multi-task learning approach needs to be developed to address these challenges.

## 3 The DeepPulmoTB dataset

### 3.1 Data sources

In DeepPulmoTB, the pulmonary CT scan images for tuberculosis are collected from the ImageCLEF TB series of tuberculosis datasets [15], [21], [14], [16]. The ImageCLEF TB is an international competition focused on tuberculosis (TB) image analysis and is part of the ImageCLEF (Image Cross-Language Evaluation Forum).^1^ ImageCLEF is a competition platform aimed at promoting cross-language image retrieval and classification technologies, including various tasks ranging from medical image analysis to the retrieval of natural images. As a standard pulmonary tuberculosis CT image dataset, the ImageCLEF TB series dataset [15], [21], [14], [16] covers cases that have been rigorously screened by medical experts and thus only includes data from patients diagnosed with pulmonary tuberculosis.

In the construction of the DeepPulmoTB dataset, to ensure data consistency and accuracy of research results, we further refined data selection criteria and clearly defined inclusion and exclusion criteria. Specifically, we focused on analyzing pulmonary tuberculosis lesions, excluding extrapulmonary tuberculosis cases from the ImageCLEF TB series datasets [15], [21], [14], [16], such as endobronchial tuberculosis and tuberculous pleurisy. As a result, we select a total of 354 chest CT scan images from different patients with pulmonary tuberculosis. Moreover, to accommodate different types of CT scan needs, our dataset includes thin and thick CT scans. Each patient's CT series consists of 125 to 512 axial projection images.

#### 3.1.1 Miliary tuberculosis

Although miliary tuberculosis affects the lungs, its pathological process usually involves the widespread distribution of micronodules in the lungs and other organs, which is significantly different from the imaging appearance of other types of tuberculosis. Because of the systemic nature of miliary TB, diagnostic and treatment strategies need to consider the entire body and not just the pulmonary manifestations. In addition, the CT imaging characteristics of miliary tuberculosis may be confused with a variety of other lung diseases, which will increase the difficulty of automatic identification and classification and may lead to a decrease in the performance of computer-aided diagnosis systems. Given these challenges and the limited number of miliary TB samples in the current dataset, we decided to exclude miliary TB from this study to ensure higher accuracy and reliability of our model in identifying and classifying the more common forms of TB.

#### 3.1.2 Endobronchial tuberculosis and tuberculous pleuritis

For endobronchial tuberculosis, it is caused by the infection of Mycobacterium tuberculosis on the epithelial cells of the trachea and endobronchial mucosa. Its primary lesions are located in the trachea and bronchi, rather than the lung parenchyma, manifesting as thickening of the tracheal and endobronchial walls and narrowing of the lumens. Secondly, tuberculous pleurisy is another form of tuberculosis infection, mainly due to Mycobacterium tuberculosis infection of the pleura (the thin membrane between the lungs and the chest wall), presenting as pleural effusion and/or thickening of pleural adhesions, also not in the lung parenchyma. However, our study focuses on pulmonary tuberculosis. Therefore, for patients with solely endobronchial tuberculosis and simple tuberculous pleurisy, since there are no lesions in the lung parenchyma, we need to exclude them. However, we did not exclude patients with endobronchial tuberculosis and tuberculous pleurisy combined with lesions in the lung parenchyma.

### 3.2 Data annotation

In the DeepPulmoTB dataset, all data are annotated by medical professionals from the Radiology Department of Universiti Putra Malaysia, ensuring the accuracy and reliability of the data. Overall, the DeepPulmoTB dataset includes two different tasks: “Lung Cavity Attribute Classification” and “Tuberculosis Multi-category Segmentation”. In the “Lung Cavity Attribute Classification” task, medical professionals annotate the number of cavities in the patient's lungs and their respective sizes, as shown in Table 2.

**Table 2:** The annotated classification of the lung cavity size and quantity in a patient's CT scan. The “Total Caver Num” column shows statistics for the number of different cavities. “No cavities” means no cavities, “1-3 cavities” means 1 to 3 cavities, and “> 3 cavities” means more than 3 cavities. The “Cavern Size” column indicates the size grouping of the cavities. “< 25 mm” indicates that the cavity size is less than 25 mm, and “> 25 mm” indicates that the cavity size is greater than or equal to 25 mm. For the “Class” column, it represents the numerical index of the classification classes.

Total Cavern Num	Cavern Size	Sample size	Class
No cavities	-	125	1
1- 3 cavities	< 25 mm	86	2
	> 25 mm	46	3
> 3 cavities	< 25 mm	52	4
	> 25 mm	45	5

In the “TB Multi-category Segmentation” task, as most of the CT images collected in the ImageCLEF series datasets are non-enhanced CTs, it is difficult for medical professionals to distinguish regions of similar density, such as the lung cavity wall and consolidation, by just observing the non-enhanced CT images. However, if the lung cavity wall and consolidation lesions are not annotated, much critical information for the diagnosis of pulmonary tuberculosis would be lost, which is detrimental to the precise diagnosis of the disease. Therefore, to provide a more comprehensive diagnosis of TB and to reduce the complexity of annotation and inconsistency in labeling, we have categorized consolidation and lung cavity wall into one semantic segmentation annotation category, namely Consolidation and Lung Cavity Wall (C-LCW). In this segmentation task, we have marked each lung CT slice image with semantic segmentation information for tuberculosis diagnosis, which includes the lung cavity, C-LCW, and the lung region, as shown in Fig. 1. We believe that this kind of segmentation annotation will better aid the model in distinguishing between normal and abnormal pulmonary tissue in images.

Moreover, in the annotation process, to ensure the quality of the annotation data, we evaluated the consistency and consistency of the segmentation and classification results between different professional doctors to obtain the annotation variability among different doctors. The concrete steps of the implementation plan are as follows:

The first step is to select multiple observers: First, we selected 3 experienced medical experts or imaging analysts as observers. Ensure that observers have the relevant background knowledge and experience to accurately understand and analyze tuberculosis lesions. The second step is to provide consistent guidance: providing consistent guidance and standardized interpretations to observers ensures that they have the same understanding and goals during the segmentation process. Describe in detail the lesion boundary definition, size judgment, etc. that should be paid attention to. The third step is to perform segmentation labeling independently: let each observer independently perform semantic segmentation and labeling on the CT image from the X-, Y-, and Z-axes. The marked software is ITK-SNAP [43]. In addition, the annotated results are output as Nifti files. In this step, we let professional doctors observe and segment without communicating with each other, to ensure that the differences in the labeling results of different experienced doctors can be observed. The fourth step involves independent attribute classification labeling for lung cavities: Each observer is responsible for categorizing attributes related to lung cavity size and quantity. The resulting classification labels are then documented in an Excel table. The fifth step is repeated observations: For each observer, we provided multiple image samples so that they could make independent segmentation observations multiple times.

**Figure 2:**
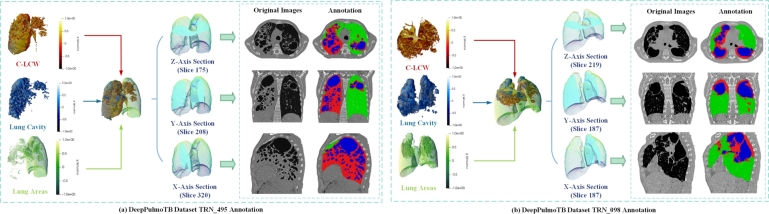
Visualization of DeepPulmoTB example data.

We visualize the results of data annotation for TRN_495 and TRN_098 in the DeepPulmoTB dataset, as shown in Fig. 2. For each example, we conducted visualizations in two dimensions: three-dimensional space and two-dimensional slices. In the three-dimensional space dimension (left module), we separately visualized the three-dimensional structures of various segmentation masks (C-LCW, lung cavities, and lung areas) within a single example from the DeepPulmoTB dataset. Additionally, we visualized the overall spatial distribution of the segmentation masks for each category within the example and displayed the positions of the spatial slices used for two-dimensional plane representation. In the two-dimensional slice dimension (right module), we visualized cross-sections along the Z, Y, and X axes.

## 4 Our approach

To demonstrate the practicality of DeepPulmoTB, we introduce a novel deep model called DeepPulmoTBNet (DPTBNet), which is capable of simultaneously performing segmentation and classification tasks.

### 4.1 Multi-task learning network

**Figure 3:**
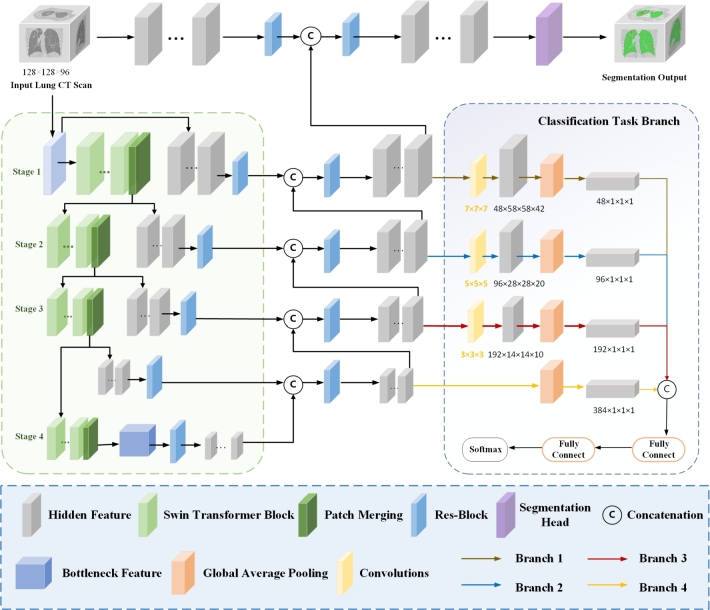
Overview of the proposed multi-task learning network.

The proposed network is shown in Fig. 3. We utilize SwinUnetR [37] as the backbone network for multi-task learning due to its great performance in 3D medical image segmentation. In the SwinUnetR backbone, the encoder part uses the Swin Transformer [24] layers to extract high-level features from the input image, preserving both local and global spatial relationships. The decoder part uses the Unetr layers to then upsample these features to the original resolution and combines them with the corresponding features from the encoder through skip connections, allowing the model to capture both high-level semantic information and detailed spatial information. To enhance the representation of TB lesion tissue features, we have increased the channel number or feature dimension size of the intermediate feature map in the model from 48 to 64.

In multi-task learning techniques, each task can extract beneficial feature information from the data of another task. This data-sharing paradigm allows the model to learn target features more effectively from limited data. In DPTBNet, we introduce an additional supervision signal and learning objective by adding a classification task module in the Decoder part for assessing the attributes of tuberculous lesion tissue. This enables the model to share knowledge and features of pulmonary tuberculosis CT images between the two tasks through the shared network's intermediate feature representations. In other words, the classification task can provide additional constraints and guidance to help the model better understand and learn the features related to TB lesions, thereby improving the performance of the semantic segmentation task. Inspired by this idea, we design DPTBNet as a multi-task learning model. DPTBNet utilizes SwinUnetR as the shared backbone network to extract common features for both classification and segmentation tasks. Specifically, we added the classification branch to the decoding part of SwinUnetR, as shown in Fig. 3. In this addition process, we first input the feature maps from branches 1, 2, 3, and 4 into the classification network. Then, we fused these shared feature maps to perform the classification task. Finally, we input the fused features into the classification branch, which consists of two fully connected (FC) layers and a softmax layer, to classify the attributes of lung cavities in the input tuberculosis CT scans [45].

### 4.2 Multi-scale feature extraction

**Figure 4:**
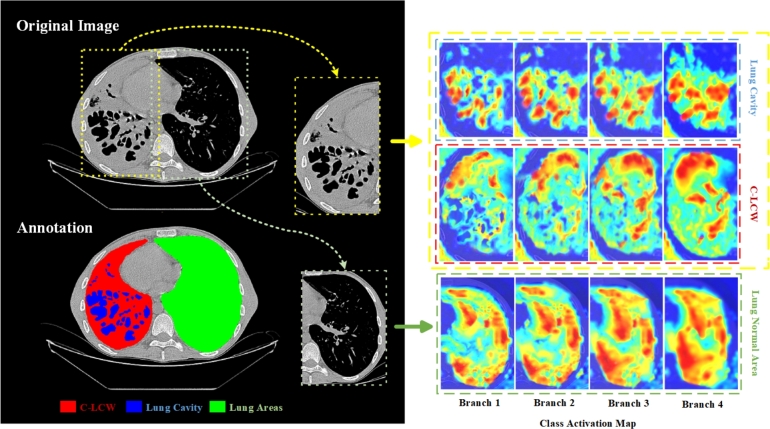
2D axial views of class activation maps from different stages of the proposed network.

To analyze the features obtained by the network at different scales, we visualized the class activation maps of branches 1 to 4 of the SwinUnetR decoder, as shown in Fig. 4. We observed that low-level features mainly capture shape and boundary information, while high-level features summarize the attributes of different targets, which are commonly used for classification tasks. However, we noticed that the distribution of pixel values for different semantic segmentation categories is imbalanced. Specifically, TB lesion tissues have fewer pixel values, causing the network to pay more attention to normal lung tissues and neglect large areas of TB lesion tissues.

To address this issue, we designed a multi-scale feature fusion model for the classification task, as shown in Fig. 2b. We connected and fused the feature maps from the four branches of SwinUnetR to obtain the classification features. For branch 1, with a feature dimension of 48×64×64×48, this dimension typically focuses on the interrelation between different targets. In this branch, we added a 7×7×7 convolutional layer to help the network capture larger spatial features and global context information, as well as to better understand the relationships between objects and overall semantics in the CT images.

For branch 2, with a feature dimension of 96×32×32×24, this dimension focuses more on the local details and relative positions of the targets. In this branch, we added a 5×5×5 convolutional layer to enhance the network's ability to capture fine structures and local contextual information, supporting the correlation between tasks and accurate target segmentation.

For branch 3, with a feature dimension of 192×16×16×12, this dimension typically focuses on local contextual information. In this branch, we added a 3×3×3 convolutional layer after the feature map. By introducing smaller convolutional kernels, the network can effectively extract local features and preserve spatial details, thereby enhancing the accuracy of semantic segmentation tasks.

For branch 4, with a feature dimension of 384×8×8×6, we did not add additional convolutional layers. Because this dimension is already suitable for capturing medium-sized features and local contextual information. This design efficiently captures medium-scale features without introducing additional complexity, providing rich feature representations for multi-task learning.

As the feature maps from different branches have different sizes and cannot be directly concatenated, we utilized Global Average Pooling (GAP) to transform the feature maps from different branches into channels of the same size. Finally, to help the network better learn complex feature representations and perform feature dimension transformation, we added two fully connected layers before the softmax function.

Overall, such a multi-scale design enables the network to effectively learn features at different scales and provides powerful feature representation for multi-task learning, thereby enhancing the performance of semantic segmentation tasks.

### 4.3 An improved version of iterative algorithm

TB lesions often exhibit complex characteristics in CT imaging. For example, the boundaries of ground-glass opacities are usually unclear, which is often caused by the presence of inflammation and exudates. However, these unclear TB lesions can increase the difficulty of segmentation. One strategy to address this issue is to use an iterative feature refinement approach. This iterative feature refinement strategy allows the model to progressively optimize segmentation results through multiple iterations, effectively utilizing previous segmentation information to guide the subsequent segmentation process. By continuously refining the feature representation, the model can better capture the boundaries and morphological information of TB lesions, thereby improving segmentation accuracy and clarity. Taking inspiration from this concept, we designed an improved version of the iterative feature refinement algorithm based on the work of [45], to further enhance the target feature-capturing capabilities of DPTBNet.

Our enhanced algorithm leverages the information from previous segmentation probability to finely adjust the input image through iterative iterations, as demonstrated in Algorithm 1. In the initial iterations, the input to the DPTBNet is tuberculosis CT scans, and the output consists of a three-dimensional probability map for segmenting tuberculosis features along with classification scores. The predicted probability map contains contextual information, guiding the model's focus on tuberculosis lesion regions, as shown in Fig. 4. In subsequent iterations, the network's input is adjusted by adding the segmentation probability map from the previous iteration. This approach enhances the model's capability to emphasize regions previously identified as potential tuberculosis lesions. By introducing this iterative feedback loop, the network can progressively adapt its features based on evolving segmentation probability maps. This mechanism strengthens the model's attention to relevant regions and further refines its predictions. The segmentation probability maps generated during each iteration are combined with the original input volume scaled by the hyperparameter *η*, striking a balance between the original and adjusted inputs. Our improvements can be summarized as follows:

**Algorithm 1:**
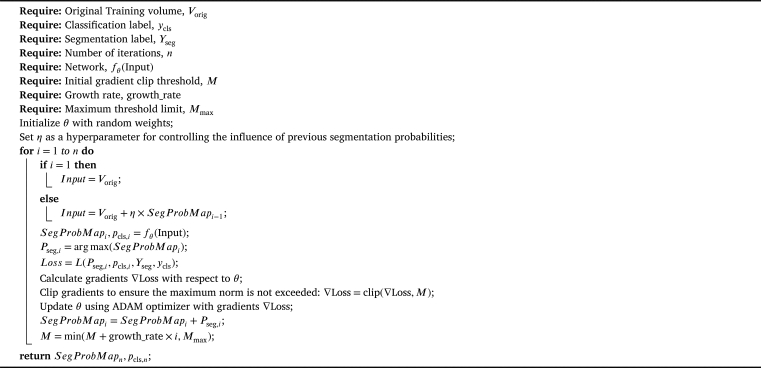
The improved iterative algorithm.

Enhanced Gradient Handling: Our algorithm introduces gradient clipping to prevent excessively large gradients during backpropagation. This is done by using the clip function on the gradients, ensuring that their maximum norm does not exceed a threshold (*M*). This addition can stabilize training and prevent gradient explosion problems.

Adaptive Learning: Our algorithm employs an adaptive approach to adjust the gradient clipping threshold (*M*). The threshold commences at an initial value and increases throughout iterations (growthrate×i) until it reaches a maximum threshold ($M_{max}$). The growth rate value is set to 0.001. This adaptive mechanism regulates the gradient clipping threshold, enhancing potential convergence and mitigating excessively aggressive clipping.

Segmentation Probability Influence: Our algorithm introduces a hyperparameter (*η*) that controls the influence of the previous segmentation probabilities on the current iteration's input. This adds a certain level of continuity between iterations by incorporating information from past segmentation probabilities.

### 4.4 Network training

The imbalance in training data categories is a common factor that can impact the learning effectiveness of a model. For instance, For example, in our DeepPulmoTB dataset, the amount of data for categories 3, 4, and 5 is much smaller than that for categories 1 and 2, as shown in Table 2. However, this data imbalance will cause the model to focus more on classes with a larger number of samples during training.

To tackle this problem, we have introduced a modified version of the Sigmoid Focal Loss [23] as the classification loss function:

(1) $$L_{\text{cls}}(P_{\text{cls}},Y_{\text{cls}})=-\frac{1}{C}\sum_{i=1}^{C}(w_{i}\cdot (Y_{{\text{cls}}_{i}}\cdot {\left(1-P_{{\text{cls}}_{i}}\right)}^{\varphi }\cdot \log(P_{{\text{cls}}_{i}})+(1-Y_{{\text{cls}}_{i}})\cdot P_{{\text{cls}}_{i}}^{\varphi }\cdot \log(1-P_{{\text{cls}}_{i}}))),$$

where $P_{cls}$ is the model's predicted probability vector, where $P_{cls_{i}}$ represents the predicted probability for the *i*-th class by the model.$Y_{cls}$ is the true label vector, where $Y_{cls_{i}}$ represents the true label for the *i*-th class. *C* denotes the total number of classes, and its value is 5. $w_{i}$ is a class weight vector, where $w_{i}$ represents the weight for the *i*-th class. $w_{i}$ is calculated as $\frac{1}{n_{i}}$. $n_{i}$ represents the number of data of the *i*-th class. *φ* is a hyperparameter used to adjust the weight of hard and easy samples in Focal Loss, and its value is set to 2.

For segmentation tasks, the pixel values of various segmentation classes are extremely imbalanced. For example, the number of pixels belonging to “lung cavity” and “C-LCW” is much smaller than that of “lung areas.” This imbalance can lead to segmentation biases. To overcome this challenge, we propose a novel segmentation loss function based on Dice loss (Milletari et al., 2016). The segmentation loss function is defined as follows:

(2) $$L_{\text{seg}}(P_{\text{seg}},Y_{\text{seg}})=1-\frac{2{\left(1-P_{\text{seg}}\right)}^{\sigma }\cdot P_{\text{seg}}\cdot Y_{\text{seg}}}{{\left(1-P_{\text{seg}}\right)}^{\sigma }\cdot P_{\text{seg}}+Y_{\text{seg}}}.$$

In this equation, we introduce a tuning factor ${\left(1-P_{\text{seg}}\right)}^{\sigma }$ to reduce the weight of easily classified samples, thus better addressing the issue of sample imbalance. This means that in semantic segmentation tasks, the loss weight for background pixels (easily classified samples) is reduced, while the loss weight for edge or minority classes (difficult-to-classify samples) is increased, facilitating better focus on challenging pixels. The introduction of ${\left(1-P_{\text{seg}}\right)}^{\sigma }$ also enhances the robustness of the loss function to slight changes in predicted probabilities. Moreover, when the predicted probability approaches 1 or 0, ${\left(1-P_{\text{seg}}\right)}^{\sigma }$ becomes very small, thereby reducing the variation of the loss function.

Finally, in our approach, we utilize a multi-task loss with a progressive trade-off factor, which can be represented as follows:

(3) $$L_{\text{joint}}=\lambda (n)L_{\text{cls}}+(1-\lambda (n))L_{\text{seg}},$$

where *n* represents the training iterations, and λ(n) is a progressive trade-off factor that changes as the training *n* increases. The λ(n) is defined as:

(4) $$\lambda (n)=\mu +\frac{\mu }{1+e^{-\mu (n-\gamma )}},$$

where *μ* is a parameter that controls the maximum and minimum values. At the upper limit of the value range of λ(n), when n→∞, the value of λ(n) will gradually tend to *μ*. That is, *μ* represents the maximum value of λ(n). *γ* is a time offset parameter, which determines the offset of the λ(n) function on the time axis. When n=γ, the value of λ(n) reaches 2*μ*. In other words, *γ* determines the location of the midpoint of λ(n), and its value is half of the total epoch. $e^{-\mu (n-\gamma )}$ is an S-shaped logistic function. When *n* is close to *γ*, $e^{-\mu (n-\gamma )}$ is close to 1, and when *n* is far away from *γ*, $e^{-\mu (n-\gamma )}$ is close to 0. This function maps values into the range [0,μ] in the form $\frac{\mu }{1+e^{-\mu (n-\gamma )}}$.

## 5 Data and experiments

### 5.1 Experiment details

To assess the efficacy of the proposed approach, we conducted a series of ablation experiments. Table 3 presents the differences among the ablation models. Specifically, we trained separate single-task models SwinUnetR_CLS_ and SwinUnetR [37] as the classification and segmentation baseline models, respectively. For SwinUnetR_CLS_, we utilized the encoding path from SwinUnetR, followed by a global average pooling (GAP) layer and two fully connected layers for the classification task [45].

**Table 3:** Ablation Models: CLS (Classification) and SEG (Segmentation). IA and IIA represent the original iterative algorithm and the improved iterative algorithm respectively. *w* and *w*/*o* represent with and without respectively. MS stands for multi-scale classification architecture. Branches 1 to 4 represent the classification branches in DPTBNet, abbreviated as B1 to B4. MS-B4 represents the exclusive use of branch 4 for classification task, with the remaining branches 1 to 3 not being utilized. This rule is also followed in the subsequent table.

Methods	IA	IIA	Branch 1	Branch 2	Branch 3	Branch 4	Multi-Scale	Task
SwinUnetR	×	×	×	×	×	×	×	SEG
SwinUnetR_CLS_	×	×	×	×	×	×	×	CLS
DPTB_*w/o* IIA, *w* MS-B4_	×	×	×	×	×	✓	×	SEG + CLS
DPTB_*w/o* IIA, *w* IA & MS-B4_	✓	×	×	×	×	✓	×	SEG + CLS
DPTB_*w* IIA & MS-B4_	×	✓	×	×	×	✓	×	SEG + CLS
DPTB_*w/o* IIA, *w* MS_	×	×	✓	✓	✓	✓	✓	SEG + CLS
DPTB_*w/o* IIA, *w* MS & IA_	✓	×	✓	✓	✓	✓	✓	SEG + CLS
DPTBNet	×	✓	✓	✓	✓	✓	✓	SEG + CLS

To explore multi-task learning, we employed the following strategies in our experiments. Firstly, we added a single-scale classification branch to SwinUnetR, utilizing the feature map from Branch 4 as the classification feature, denoted as DPTB_*w*/*o* IIA, *w* MS-B4_. Subsequently, we trained DPTB_*w*/*o* IIA, *w* MS-B4_ using the improved iterative algorithm (IIA) with n=3 iterations denoted as DPTB_*w* IIA & MS-B4_. Additionally, for comparison with the original iterative algorithm (IA) [45], we trained DPTB_*w*/*o* IIA, *w* MS-B4_ with n=2 iterations, denoted as DPTB_*w*/*o* IIA, *w* IA & MS-B4_.

Furthermore, we explored the use of the proposed multi-scale classification branch presented in Fig. 3 instead of the single-scale classification branch for multi-task learning, denoted as DPTB_*w*/*o* IIA, *w* MS_. We then trained the DPTB_*w*/*o* IIA, *w* MS_ model using the improved iterative feature-refining algorithm with n=3 iterations, denoted as DPTBNet. For comparison, we also trained DPTB_*w*/*o* IIA, *w* MS_ using the original iterative feature-refining algorithm [45] with n=2 iterations, denoted as DPTB_*w*/*o* IIA, *w* MS & IA_.

To assess the effectiveness of our proposed method, we conducted a comparative study with existing multi-task learning approaches (as described in Section 2.5), including the methods proposed by [2], [45], [35], and [11]. As some of these methods were originally designed for 2D operations, we extended them to 3D operations to ensure a fair comparison. To maintain consistency, we replaced the backbone networks used in these methods with the same SwinUnetR backbone utilized in our proposed method. For the evaluation, we performed five-fold cross-validation using the DeepPulmoTB dataset and employed the proposed loss function from Eq. (3) in the experiments. The other parameters of the compared methods were kept consistent with the values reported in the respective works by [2], [45], [35], and [11]. This ensured a fair and unbiased comparison among the methods under investigation.

In data preprocessing, we leveraged data augmentation modules from the MONAI (Medical Open Network for AI) [5] library to increase data diversity and improve model generalization. The specific parameter configurations were as follows: For input images, we normalized the pixel values to the range of 0 to 1, ensuring uniform data scaling. To enhance data variability, we utilized the RandCropByPosNegLabeld module for random cropping of input images and labels. The crop size was set as (128,128,96) with one crop per sample. For further augmentation, we applied the RandFlipd module, enabling random flipping of input images and labels along the specified axes (x, y, z). To introduce additional variations, the RandRotate90d module was used, allowing for random rotations of input images and labels on all three axes, with up to 3 rotations of 90 degrees each. To augment pixel intensities, the RandShiftIntensityd module was utilized, applying random pixel intensity shifts to input images, which aids in improving model robustness during training.

### 5.2 Quantitative evaluation metrics

In our analysis, we employed the Dice Similarity Coefficient (DSC), Hausdorff Distance at the 95th percentile (95HD voxel), and Intersection over Union (IoU) as the principal quantitative metrics to assess the segmentation fidelity for tuberculosis lesions and normal pulmonary structures:

The Hausdorff Distance, denoted as H(A,B), delineates the extent of divergence between two sets of points, *A* and *B*, and is computed as follows:

(5) $$H(A,B)=\max\left\{\underset{a\in A}{\sup}\underset{b\in B}{\inf}d(a,b),\underset{b\in B}{\sup}\underset{a\in A}{\inf}d(b,a)\right\}$$

where d(a,b) signifies the metric distance, typically the Euclidean, between point *a* from set *A* and point *b* from set *B*, with sup and inf representing the supremum and infimum over the distances, respectively.

To mitigate outlier sensitivity inherent in the Hausdorff Distance, this paper considers the 95HD metric, which isolates the 95th percentile within the cumulative distribution of the distances, thus providing a more robust measure of segmentation accuracy:

(6) $$95\text{HD (voxel)}=P_{95}\left\{d(a,B)|a\in A\right\}\cup \left\{d(b,A)|b\in B\right\}$$

In addition, the Dice Similarity Coefficient (DSC) and Intersection over Union (IoU) are also the key metrics that evaluate the segmentation accuracy between the model-predicted region and the ground truth. The DSC is calculated as twice the intersection of *A* and *B*, normalized by the sum of their sizes:

(7) $$DSC=\frac{2\times |A\cap B|}{\left|A|+|B\right|}$$

Conversely, the IoU metric offers a ratio of the intersection to the union of the two sets:

(8) $$IoU=\frac{\left|A\cap B\right|}{\left|A\cup B\right|}$$

where *A* and *B* correspond to the pixel or voxel sets within the ground truth and the algorithmically predicted segmentation contours, respectively.

For the quantitative evaluation of lung cavity attributes classification, we utilized receiver operating characteristic (ROC) curves, area under the ROC curve (AUC), recall (REC), precision (PRE), accuracy (ACC), false positive rate (FPR), and F1-score (F1).

(9) $$\text{ACC}=\frac{\text{TP}+\text{TN}}{\text{TP}+\text{TN}+\text{FP}+\text{FN}},$$

(10) $$\text{REC}=\frac{\text{TP}}{\text{TP}+\text{FN}},$$

(11) $$\text{PRE}=\frac{\text{TP}}{\text{TP}+\text{FP}},$$

(12) $$\text{FPR}=\frac{\text{FP}}{\text{FP}+\text{TN}},$$

(13) $$\text{F1}=2\times \frac{\text{PRE}\times \text{REC}}{\text{PRE}+\text{REC}},$$

where FP, TP, FN, and TN represent the counts of false positives, true positives, false negatives, and true negatives, respectively.

## 6 Results

### 6.1 Tuberculosis lesions and lung areas segmentation

To investigate the optimal performance of the improved iterative feature refinement algorithm under different strategies, we trained the DPTBNet with varying numbers of iterations (*n*) and weight parameters (*η*) and summarized the results in Table 4. From the table, we observed that the iterative training strategy shows the best performance when η=0.7 and n=3, achieving 95HD, DSC, IoU, and values of 14.24, 69.6% and 55.8%, respectively. This outcome suggests that with this particular combination of variables, the model can effectively use the iterative feature refinement method by appropriately combining the spatial information from the original training volume and the semantic information from the segmentation probability map, resulting in more accurate pixel-level segmentation. The comparative analysis of different *η* values highlighted that tuning the weight parameter *η* to 0.7 in the IIA can achieve a superior equilibrium between the original training volume and the segmentation probability map. This, in turn, leads to improved algorithmic performance when contrasted with scenarios where this parameter remains unadjusted (η=1). As a result, for all ensuing experiments, we have opted to consistently set η=0.7 and n=3.

**Table 4:** Segmentation performance of DPTBNet with respect to the weight parameters (*η*) and numbers of iterations (*N*). *SD* stands for Standard Deviation. This rule is also followed in the subsequent table.

n	*η* = 0.1 ( ± SD)			*η* = 0.4 ( ± SD)			*η* = 0.7 ( ± SD)			*η* = 1 ( ± SD)		
	95HD (voxel)	IoU (%)	DSC (%)	95HD (voxel)	IoU (%)	DSC (%)	95HD (voxel)	IoU (%)	DSC (%)	95HD (voxel)	IoU (%)	DSC (%)
0	25.19 ± 4.67	39.5 ± 10.5	53.9 ± 11.8	25.43 ± 8.12	40.4 ± 14.2	53.3 ± 15.6	22.62 ± 3.64	46.4 ± 7.8	59.3 ± 8.4	24.13 ± 6.67	43.8 ± 10.5	56.9 ± 12.6
1	22.28 ± 5.89	46.9 ± 12.4	59.9 ± 12.3	19.69 ± 4.14	48.1 ± 9.6	61.9 ± 10.8	17.13 ± 7.86	51.6 ± 12.6	65.9 ± 12.4	17.21 ± 5.53	50.1 ± 16.2	65.1 ± 12.9
2	21.79 ± 3.65	47.2 ± 8.9	60.4 ± 9.1	18.43 ± 5.42	50.4 ± 12.9	63.4 ± 13.7	16.04 ± 6.32	52.6 ± 13.5	67.4 ± 15.4	16.82 ± 4.78	53.4 ± 10.2	66.5 ± 14.3
3	18.92 ± 3.91	49.4 ± 9.6	62.2 ± 11.7	15.96 ± 4.83	53.6 ± 11.9	66.2 ± 10.4	**14.24** ± **5.38**	**55.8** ± **13.9**	**69.6** ± **15.2**	14.53 ± 5.89	55.3 ± 9.2	68.2 ± 12.8
4	20.61 ± 6.57	48.7 ± 12.8	61.9 ± 14.3	17.02 ± 3.67	54.6 ± 10.7	65.9 ± 10.5	14.98 ± 4.16	55.2 ± 13.7	68.1 ± 14.2	15.16 ± 3.78	54.2 ± 6.4	67.3 ± 7.1

Table 5 presents the quantitative results of ablative experiments based on five-fold cross-validation. For the segmentation task, we observed that our DPTBNet model outperforms other methods in all metrics, with 95HD, DSC, and IoU values of 14.24, 69.6%, and 55.8%, respectively. By comparing the results of DPTB_*w*/*o* IIA, *w* MS-B4_ and SwinUnetR, we observe that DPTB_*w*/*o* IIA, *w* MS-B4_ performs better on the segmentation task, confirming the beneficial impact of incorporating classification information into the segmentation task.

**Table 5:** Segmentation and classification task performance of ablation models.

Methods	Segmentation ( ± SD)			Classification				
	95HD (voxel)	IoU (%)	DSC (%)	ACC	REC	PRE	FPR	F1
SwinUnetR	23.06 ± 7.27	46.9 ± 12.7	58.9 ± 11.9	-	-	-	-	-
SwinUnetR_CLS_	-	-	-	0.578	0.697	0.667	0.698	0.681
DPTB_*w*/*o* IIA, *w* MS-B4_	21.25 ± 4.87	49.3 ± 11.3	60.4 ± 13.2	0.641	0.762	0.682	0.617	0.718
DPTB_*w*/*o* IIA, *w* IA & MS-B4_	17.01 ± 3.62	52.0 ± 14.1	65.7 ± 12.0	0.648	0.768	0.696	0.610	0.729
DPTB_*w* IIA & MS-B4_	14.99 ± 3.98	55.0 ± 12.9	68.2 ± 17.2	0.723	0.813	0.781	0.553	0.796
DPTB_*w*/*o* IIA, *w* MS_	19.43 ± 5.46	49.2 ± 15.7	62.3 ± 12.3	0.711	**0.823**	0.773	0.531	0.797
DPTB_*w*/*o* IIA, *w* MS & IA_	15.87 ± 4.57	53.7 ± 12.6	66.9 ± 14.1	0.738	0.772	0.812	0.386	0.791
DPTBNet	**14.24** ± **5.38**	**55.8** ± **13.9**	**69.6** ± **15.2**	**0.757**	0.804	**0.817**	**0.408**	**0.810**

In contrast to DPTB_*w*/*o* IIA, *w* MS-B4_, DPTB_*w*/*o* IIA, *w* MS_ adopts multi-scale feature extraction for classification and exhibits better performance than DPTB_*w*/*o* IIA, *w* MS-B4_ and SwinUnetR on the segmentation task. Furthermore, from Table 5, we observed that our improved iterative algorithm enhances the segmentation performance compared to the original iterative algorithm. For instance, DPTBNet and DPTB_*w* IIA & MS-B4_ achieve DSC improvements of 7.3% and 7.8%, respectively, compared to DPTB_*w*/*o* IIA, *w* MS_ and DPTB_*w*/*o* IIA, *w* MS-B4_. Moreover, both the improved and original iterative feature refinement strategies enhance the segmentation performance. For example, DPTBNet and DPTB_*w*/*o* IIA, *w* MS & IA_achieve DSC improvements of 4.6% and 2.7%, respectively, compared to DPTB_*w*/*o* IIA, *w* MS_.

**Figure 5:**
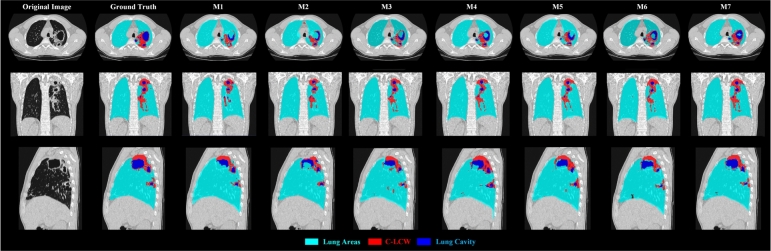
Segmentation masks predicted by various ablation models. M1-M7 correspond to SwinUnetR, DPTB_*w/o* IIA, *w* MS-B4_, DPTB_*w/o* IIA, *w* IA & MS-B4_, DPTB_*w* IIA & MS-B4_, DPTB_*w/o* IIA, *w* MS_, DPTB_*w/o* IIA, *w* MS & IA_ and DPTBNet, respectively.

Table 6 lists the segmentation performance of each class mask in the semantic segmentation task based on five-fold cross-validation. From the table, we observed that the DPTBNet variants outperform the baseline model SwinUnetR in three classes (lung cavity, C-LCW, and lung areas) in terms of 95HD, IoU, and DSC, demonstrating the effectiveness and superior performance of DPTBNet series models. In addition, Fig. 5 displays the three cross-sectional views of TB lesions and normal lung areas. From the figure, we observed that the results obtained from our method align well with ground truth in three dimensions compared to the other methods.

**Table 6:** The segmentation performance of each class mask in the semantic segmentation task based on five-fold cross-validation.

Methods		Lung Cavity ( ± SD)			C-LCW ( ± SD)			Lung Areas ( ± SD)		
		95HD (voxel)	IoU (%)	DSC (%)	95HD (voxel)	IoU (%)	DSC (%)	95HD (voxel)	IoU (%)	DSC (%)
Fold 1	SwinUnetR	40.53 ± 10.62	21.4 ± 17.1	34.9 ± 18.4	25.14 ± 8.45	38.0 ± 13.8	51.3 ± 12.9	5.99 ± 2.81	68.4 ± 9.3	80.6 ± 9.7
	DPTB_*w*/*o* IIA, *w* MS-B4_	37.28 ± 9.64	23.6 ± 18.3	37.0 ± 19.1	23.59 ± 11.72	41.8 ± 14.3	53.4 ± 15.5	4.76 ± 2.47	72.0 ± 5.2	84.0 ± 6.3
	DPTB_*w*/*o* IIA, *w* IA & MS-B4_	33.17 ± 7.13	29.2 ± 9.5	44.0 ± 12.6	13.48 ± 9.84	52.2 ± 18.1	67.0 ± 16.4	3.52 ± 1.35	76.9 ± 9.2	90.3 ± 9.7
	DPTB_*w* IIA & MS-B4_	30.72 ± 8.01	32.5 ± 15.6	44.2 ± 12.9	**9.76** ± **8.52**	**56.6** ± **12.4**	**69.7** ± **13.2**	2.37 ± 1.02	79.7 ± 8.9	93.2 ± 9.8
	DPTB_*w*/*o* IIA, *w* MS_	33.28 ± 9.61	27.9 ± 14.4	40.1 ± 15.3	17.72 ± 9.76	47.4 ± 15.8	56.9 ± 14.5	4.21 ± 1.64	73.3 ± 9.3	84.4 ± 8.3
	DPTB_*w*/*o* IIA, *w* MS & IA_	30.79 ± 8.53	31.2 ± 14.5	43.2 ± 12.1	11.23 ± 7.43	54.2 ± 16.5	68.3 ± 15.9	2.96 ± 0.58	78.9 ± 6.1	92.7 ± 5.5
	DPTBNet	**28.47** ± **8.37**	**33.6** ± **15.7**	**46.9** ± **16.1**	9.87 ± 9.65	56.3 ± 12.3	69.5 ± 13.4	**2.74** ± **1.12**	**80.1** ± **6.8**	**93.8** ± **7.7**

Fold 2	SwinUnetR	36.65 ± 11.73	24.2 ± 14.2	36.3 ± 17.4	21.97 ± 10.67	41.6 ± 14.6	54.1 ± 15.9	4.46 ± 1.67	73.1 ± 5.6	86.8 ± 6.1
	DPTB_*w*/*o* IIA, *w* MS-B4_	34.21 ± 7.87	26.9 ± 14.5	38.5 ± 16.2	20.64 ± 9.14	44.0 ± 13.7	56.6 ± 12.8	4.04 ± 2.04	75.7 ± 7.1	89.5 ± 5.8
	DPTB_*w*/*o* IIA, *w* IA & MS-B4_	30.87 ± 9.68	32.7 ± 18.8	42.1 ± 18.4	13.28 ± 8.37	51.1 ± 14.2	64.4 ± 15.7	3.43 ± 0.96	76.9 ± 6.7	90.9 ± 7.6
	DPTB_*w* IIA & MS-B4_	27.38 ± 9.81	35.3 ± 13.2	47.2 ± 14.7	11.60 ± 5.05	53.7 ± 9.4	66.8 ± 11.5	**2.87** ± **1.26**	**79.9** ± **6.9**	**93.4** ± **6.2**
	DPTB_*w*/*o* IIA, *w* MS_	32.63 ± 7.62	28.2 ± 13.5	40.1 ± 15.1	18.79 ± 8.54	46.3 ± 14.7	58.2 ± 16.3	4.23 ± 2.11	75.9 ± 7.4	91.2 ± 8.8
	DPTB_*w*/*o* IIA, *w* MS & IA_	28.14 ± 10.78	34.0 ± 12.4	43.1 ± 15.9	12.32 ± 5.73	52.3 ± 14.6	66.0 ± 11.8	3.03 ± 1.26	78.7 ± 6.2	92.2 ± 7.5
	DPTBNet	**26.81** ± **6.56**	**36.3** ± **11.9**	**52.4** ± **13.8**	**10.32** ± **7.35**	**54.8** ± **12.6**	**67.5** ± **13.3**	2.89 ± 1.87	**79.9** ± **5.4**	**93.4** ± **6.1**

Fold 3	SwinUnetR	41.24 ± 6.57	17.1 ± 9.3	33.2 ± 13.1	26.14 ± 7.73	34.9 ± 12.6	49.2 ± 11.9	5.16 ± 2.17	71.0 ± 6.7	82.9 ± 5.8
	DPTB_*w*/*o* IIA, *w* MS-B4_	39.72 ± 10.75	19.5 ± 14.2	35.5 ± 14.9	23.42 ± 8.64	40.2 ± 12.3	51.2 ± 15.4	4.12 ± 1.15	74.5 ± 5.5	86.1 ± 7.1
	DPTB_*w*/*o* IIA, *w* IA & MS-B4_	33.69 ± 9.02	21.2 ± 12.1	43.7 ± 13.6	18.69 ± 9.56	46.8 ± 13.1	58.7 ± 12.9	3.84 ± 2.16	77.6 ± 6.2	90.7 ± 7.1
	DPTB_*w* IIA & MS-B4_	32.63 ± 9.16	24.4 ± 13.5	44.2 ± 13.9	15.47 ± 9.37	49.0 ± 16.3	62.9 ± 15.1	2.98 ± 2.23	79.0 ± 6.4	93.3 ± 6.8
	DPTB_*w*/*o* IIA, *w* MS_	35.47 ± 10.21	21.9 ± 12.7	37.7 ± 14.4	23.89 ± 11.45	42.1 ± 15.2	52.1 ± 16.8	3.38 ± 2.17	76.7 ± 7.3	89.2 ± 7.5
	DPTB_*w*/*o* IIA, *w* MS & IA_	33.12 ± 7.82	23.7 ± 13.9	43.0 ± 15.6	16.92 ± 6.75	48.6 ± 8.6	59.8 ± 10.1	2.92 ± 1.89	79.2 ± 6.5	93.9 ± 7.4
	DPTBNet	**31.75** ± **7.71**	**26.5** ± **12.7**	**45.0** ± **14.6**	**13.68** ± **6.08**	**50.8** ± **12.9**	**63.1** ± **15.1**	**2.27** ± **1.02**	**81.1** ± **7.2**	**94.2** ± **6.7**

Fold 4	SwinUnetR	39.85 ± 11.83	22.5 ± 16.9	35.4 ± 18.2	20.98 ± 10.34	43.4 ± 12.7	56.9 ± 14.3	4.92 ± 2.04	71.4 ± 6.9	84.3 ± 6.4
	DPTB_*w*/*o* IIA, *w* MS-B4_	35.37 ± 9.67	25.9 ± 12.1	39.0 ± 16.5	18.72 ± 7.59	45.9 ± 12.5	58.6 ± 13.8	4.03 ± 0.96	75.8 ± 8.3	87.0 ± 7.2
	DPTB_*w*/*o* IIA, *w* IA & MS-B4_	29.23 ± 4.31	33.0 ± 8.3	44.8 ± 9.6	13.87 ± 4.12	51.1 ± 8.7	64.9 ± 11.8	3.71 ± 1.32	78.0 ± 7.6	89.0 ± 6.7
	DPTB_*w* IIA & MS-B4_	27.14 ± 8.32	36.2 ± 13.4	46.4 ± 17.2	12.12 ± 8.63	53.9 ± 13.5	65.7 ± 12.9	2.62 ± 0.86	80.4 ± 6.1	93.9 ± 6.5
	DPTB_*w*/*o* IIA, *w* MS_	33.72 ± 3.08	27.0 ± 8.6	40.6 ± 11.3	15.92 ± 9.67	48.7 ± 14.9	60.2 ± 13.6	3.45 ± 1.98	76.3 ± 7.7	90.1 ± 8.9
	DPTB_*w*/*o* IIA, *w* MS & IA_	29.16 ± 5.98	33.5 ± 10.9	46.3 ± 13.7	11.98 ± 4.53	53.2 ± 15.8	65.6 ± 16.3	2.89 ± 1.45	79.3 ± 6.6	93.9 ± 7.7
	DPTBNet	**26.92** ± **5.17**	**36.2** ± **14.5**	**50.3** ± **15.4**	**10.17** ± **6.79**	**55.4** ± **13.7**	**68.8** ± **14.1**	**2.12** ± **1.13**	**81.3** ± **7.3**	**94.1** ± **8.2**

Fold 5	SwinUnetR	42.72 ± 10.28	22.1 ± 15.3	31.5 ± 16.7	25.98 ± 11.55	36.6 ± 15.3	50.5 ± 16.9	4.13 ± 2.10	71.4 ± 9.1	86.1 ± 8.5
	DPTB_*w*/*o* IIA, *w* MS-B4_	40.38 ± 6.67	24.5 ± 8.6	34.2 ± 10.1	24.83 ± 7.68	37.8 ± 13.5	52.8 ± 16.2	3.72 ± 1.38	74.2 ± 7.9	89.5 ± 7.4
	DPTB_*w*/*o* IIA, *w* IA & MS-B4_	33.42 ± 5.17	31.0 ± 11.9	41.1 ± 14.2	17.97 ± 9.73	44.1 ± 16.8	58.2 ± 12.3	2.86 ± 1.84	78.4 ± 6.5	94.8 ± 7.3
	DPTB_*w* IIA & MS-B4_	**28.34** ± **6.12**	**34.9** ± **12.5**	**45.6** ± **14.8**	16.74 ± 9.31	47.9 ± 15.1	59.5 ± 15.3	2.08 ± 1.25	80.1 ± 5.4	95.0 ± 6.7
	DPTB_*w*/*o* IIA, *w* MS_	37.87 ± 5.43	27.4 ± 13.1	36.2 ± 15.2	23.74 ± 7.84	39.6 ± 13.4	54.1 ± 16.1	3.12 ± 1.64	76.8 ± 7.9	91.7 ± 6.8
	DPTB_*w*/*o* IIA, *w* MS & IA_	30.78 ± 6.65	33.5 ± 14.8	42.4 ± 13.9	18.97 ± 6.73	46.3 ± 10.1	58.4 ± 11.7	2.81 ± 1.17	79.5 ± 7.3	94.3 ± 6.6
	DPTBNet	29.62 ± 7.53	33.6 ± 13.2	44.9 ± 15.7	**13.98** ± **8.47**	**49.3** ± **14.4**	**62.2** ± **15.8**	**1.98** ± **0.85**	**81.7** ± **7.9**	**95.2** ± **7.1**

### 6.2 Classification of lung cavity attributes

**Figure 6:**
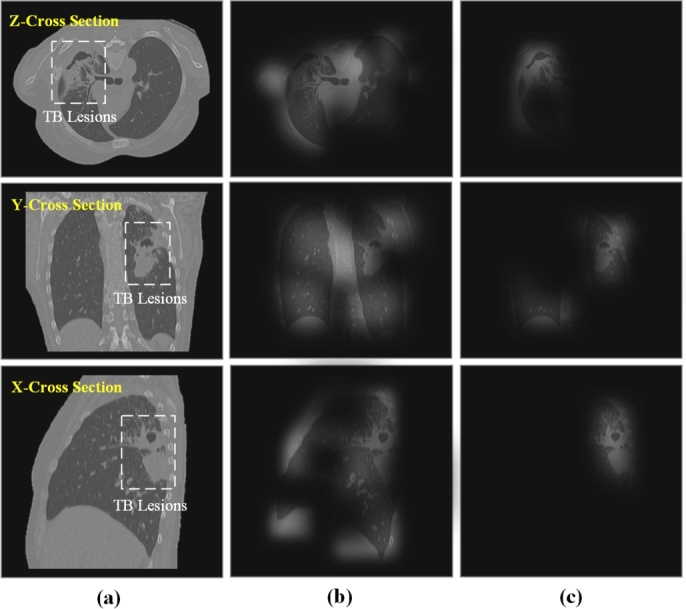
Comparison of the focus areas between single-Task (classification) and multi-Task learning networks for classification tasks. (a) - (c) respectively represent the original image, class activation maps of SwinUnetR_CLS_, and class activation maps of DPTB_*w*/*o* IIA, *w* MS_.

Fig. 6 demonstrates a comparative analysis of the focus areas between a single-task classification network and a multi-task learning network. We observed that compared to SwinUnetR_CLS_, DPTB_*w*/*o* IIA, *w* MS_, which integrates multi-scale segmentation feature maps, is more effective in capturing the TB lesion feature regions.

Table 5 also summarizes the quantitative classification results of single-classification and multi-task learning models. DPTB_*w*/*o* IIA *w* MS-B4_ employs single-scale feature extraction for classification and demonstrates an improvement in classification results compared to the classification baseline model, SwinUnetR_CLS_, confirming the enhancement brought by segmentation to classification. Moreover, from the table, we observed that DPTB_*w*/*o* IIA, *w* MS_ achieved the best REC value of 0.823, while DPTBNet attained optimal values of 0.757, 0.817, 0.408, and 0.810 for ACC, PRE, FPR, and F1 scores, respectively.

**Figure 7:**
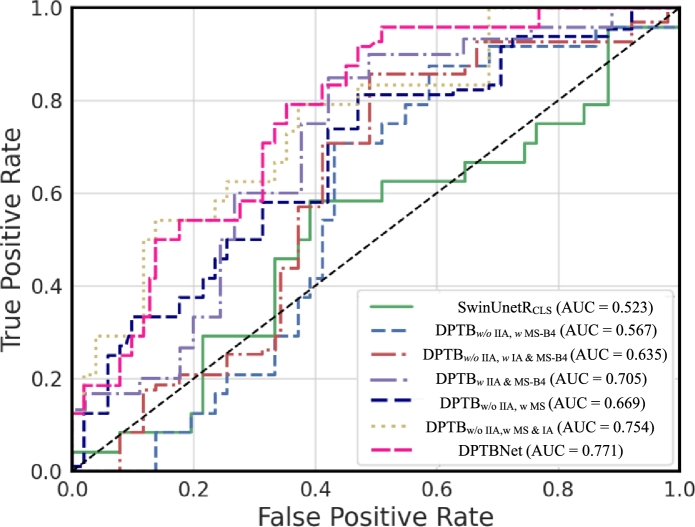
ROCs of ablation models.

Fig. 7 displays the ROC curves for each ablation model. In comparison to DPTB_*w* IIA & MS-B4_, DPTBNet exhibited superior performance by elevating the AUC from 0.705 to 0.771. Moreover, we observed that the AUC values of our proposed improved iterative algorithm are better than the original iterative algorithm, whether in a single-scale or multi-scale network structure.

To further compare the impact of feature map combinations from different scales on the classification task, we trained DPTBNet with various feature combinations, as shown in Table 7. DPTB-Bi-j connected the feature maps from the i-th to j-th branches of DPTBNet. For instance, DPTB-B1-3 utilized the feature maps from branches 1, 2, and 3. From the table, we observed that the fusion of features from branches 1 to 4 yielded the optimal values of 0.711, 0.773, 0.475, and 0.797 for ACC, PRE, FPR, and F1 scores, respectively.

**Table 7:** Evaluation of classification performance using different multi-scale features.

Methods	IA	IIA	Branch 1	Branch 2	Branch 3	Branch 4	Multi-Scale	ACC	REC	PRE	FPR	F1
DPTB-B1	×	×	✓	×	×	×	×	0.663	0.756	0.716	0.583	0.735
DPTB-B1-2	×	×	✓	✓	×	×	✓	0.697	0.773	0.738	0.526	0.755
DPTB-B1-3	×	×	✓	✓	✓	×	✓	0.708	**0.829**	0.754	0.492	0.790
DPTB-B1-4	×	×	✓	✓	✓	✓	✓	**0.711**	0.823	**0.773**	**0.475**	**0.797**

### 6.3 Multi-task learning

The purpose of the hyperparameter *σ* in Eq. (2) is to adjust the weighting of the overlap between predicted results and true labels in the loss function, thus influencing the degree of adjustment to model predictions during the training process. In the proposed DPTBNet model, the performance of multi-task learning based on different *σ* values is listed in Table 8. We observed that when *σ* is set to 2, DPTBNet achieves the best performance, and it performs the best in terms of 95HD, IoU, DSC, ACC, FPR, and F1 scores. Although the classification REC and PRE performance reach their best values of 0.813 and 0.820 when σ=3 and σ=0, the segmentation performance is worse than that of σ=2. Among them, σ=0 represents the original dice loss.

**Table 8:** The effects of different values of *σ* on multi-task learning.

*σ*	Segmentation ( ± SD)			Classification				
	95HD (voxel)	IoU (%)	DSC (%)	ACC	REC	PRE	FPR	F1
0	14.53 ± 4.89	54.6 ± 13.2	68.3 ± 12.3	0.739	0.785	**0.820**	0.462	0.802
1	22.76 ± 3.98	47.2 ± 11.7	59.3 ± 11.2	0.712	0.796	0.791	0.580	0.793
2	**14.24** ± **5.38**	**55.8** ± **13.9**	**69.6** ± **15.2**	**0.757**	0.804	0.817	**0.408**	**0.810**
3	14.98 ± 6.13	55.0 ± 14.8	68.2 ± 13.7	0.723	**0.813**	0.802	0.557	0.807
4	24.08 ± 4.95	45.2 ± 12.3	57.8 ± 14.5	0.641	0.762	0.732	0.617	0.746

The purpose of Eq. (3) and (4) is to balance the performance of classification and semantic segmentation in multi-task learning, where the hyperparameter *μ* is used to control the maximum and minimum values. In the proposed DPTBNet model, the performance of classification and semantic segmentation based on different *μ* values is listed in Table 9. From the table, we observed that when *μ* is set to 0.3, DPTBNet achieves the best balance between classification loss and semantic segmentation loss, and it performs the best in terms of 95HD, IoU, DSC, ACC, PRE, FPR, and F1 scores.

**Table 9:** The effects of different values of *μ* on multi-task learning.

*μ*	Segmentation ( ± SD)			Classification				
	95HD (voxel)	IoU (%)	DSC (%)	ACC	REC	PRE	FPR	F1
0.1	16.88 ± 6.17	53.9 ± 14.2	66.9 ± 14.3	0.693	0.748	0.769	0.677	0.758
0.3	**14.24** ± **5.38**	**55.8** ± **13.9**	**69.6** ± **15.2**	**0.757**	0.804	**0.817**	**0.408**	**0.810**
0.5	15.03 ± 4.78	54.7 ± 11.5	68.7 ± 12.7	0.723	**0.831**	0.789	0.492	0.809
0.7	16.94 ± 5.12	52.3 ± 12.6	66.8 ± 11.5	0.708	0.763	0.804	0.571	0.783
0.9	16.78 ± 4.64	53.9 ± 12.1	67.3 ± 13.8	0.719	0.792	0.815	0.632	0.803

Therefore, in our experiments, we set σ=0.8 and μ=0.3. We compared the proposed method with other multi-task learning methods, and the comparison results are summarized in Table 10. We trained four existing methods on the DeepPulmoTB dataset. Table 10 shows that the proposed DPTBNet model achieves the best performance in all metrics except PRE, and DPTBNet obtains the second-best PRE score among all methods.

**Table 10:** Comparison with existing multi-task learning methods.

Methods	Segmentation ( ± SD)			Classification				
	95HD (voxel)	IoU (%)	DSC (%)	ACC	REC	PRE	FPR	F1
[11]	29.36 ± 7.64	38.8 ± 15.8	52.4 ± 16.7	0.675	0.717	0.732	0.672	0.724
[35]	25.17 ± 5.78	42.6 ± 12.7	57.7 ± 12.9	0.692	0.731	0.756	0.629	0.743
[45]	19.95 ± 6.31	50.2 ± 14.3	62.3 ± 14.1	0.713	0.755	0.812	0.534	0.782
[2]	18.76 ± 5.57	51.4 ± 12.5	64.1 ± 13.6	0.728	0.763	**0.858**	0.463	0.807
DPTBNet	**14.24** ± **5.38**	**55.8** ± **13.9**	**69.6** ± **15.2**	**0.757**	**0.804**	0.817	**0.408**	**0.810**

## 7 Conclusion

In this paper, we present DeepPulmoTB, an innovative CT multi-task learning dataset specifically curated for tuberculosis (TB) diagnosis. In contrast to the previous TB lesion tissue recognition dataset, DeepPulmoTB stands out as the first multi-task learning dataset capable of simultaneously conducting TB multi-category semantic segmentation and lung cavity attributes classification tasks. To highlight the benefits of this multi-task learning approach for TB, we introduce a novel model called DeepPulmoTBNet (DPTBNet). This model can address two tasks concurrently: the segmentation of lesion tissue and the classification of CT images. To further enhance the model's ability to capture TB lesion features, we propose an improved iterative optimization algorithm. This algorithm intricately refines feature maps by integrating probability maps from previous iterations. Experimental results show that DPTBNet provides better results in TB segmentation and lung cavity attributes classification than a single-task model trained separately, achieving state-of-the-art performance among existing methods.

## CRediT authorship contribution statement

**Zhuoyi Tan:** Conceptualization, Data curation, Formal analysis, Investigation, Methodology, Project administration, Resources, Software, Validation, Visualization, Writing – original draft, Writing – review & editing. **Hizmawati Madzin:** Data curation, Funding acquisition, Investigation, Methodology, Software, Supervision. **Bahari Norafida:** Conceptualization, Data curation, Methodology. **Yang ChongShuang:** Data curation, Formal analysis, Validation. **Wei Sun:** Project administration, Resources, Software, Validation. **Tianyu Nie:** Investigation, Visualization. **Fengzhou Cai:** Methodology, Writing – review & editing.

## Declaration of Competing Interest

The authors declare the following financial interests/personal relationships which may be considered as potential competing interests:

Zhuoyi Tan reports financial support was provided by Malaysia Ministry of Higher Education and Universiti Putra Malaysia.

## Data Availability

The DeepPulmoTB dataset will be made available at https://github.com/SupCodeTech/DeepPulmoTB.

## Footnotes

1 https://www.imageclef.org/.
